# Supplementary material for: Ion desolvation for boosting the charge storage performance in Ti3C2 MXene electrode
Source: Nat Commun. 2025 Apr 23;16:3813. doi: 10.1038/s41467-025-58700-x (PMC12018951; doi:10.1038/s41467-025-58700-x)
Supplement: Supplementary file 1 — Supplementary Information [file 41467_2025_58700_MOESM1_ESM.pdf]

Supplementary Information for

Ion desolvation for boosting the charge storage performance in Ti_3C_2 MXene electrode

Zheng Bo[1], Rui Wang[1], Bin Wang[2], Sanjay Sunny[3], Yuping Zhao[4], Kangkang Ge[3], Kui Xu[5], Yajing Song[4], Encarnacion Raymundo-Piñero[6], Zifeng Lin[2], Hui Shao[7], Qian Yu[4]*, Jianhua Yan[1]*, Kefa Cen[1], Pierre-Louis Taberna[3, 8], Patrice Simon[3, 8]*

[1]State Key Laboratory of Clean Energy Utilization, College of Energy Engineering, Zhejiang University, Hangzhou, Zhejiang 310027, China.

[2]College of Materials Science and Engineering, Sichuan University, Chengdu 610065, China.

[3]Université Paul Sabatier, CIRIMAT UMR CNRS 5085, 118 Route de Narbonne, 31062 Toulouse, France.

[4]Center of Electron Microscopy and State Key Laboratory of Silicon and Advanced Semiconductor Materials, School of Materials Science and Engineering, Zhejiang University, Hangzhou, 310027, China.

[5]School of Flexible Electronics (Future Technologies) & Institute of Advanced Materials (IAM), Nanjing Tech University (Nanjing Tech),30 South Puzhu Road, Nanjing 211816, P.R. China.

[6]Université Orléans, CNRS, CEMHTI UPR3079, Orléans, France.

[7]i-Lab, CAS Center for Excellence in Nanoscience, Suzhou Institute of Nano-Tech and Nano-Bionics (SINANO), Chinese Academy of Sciences (CAS), Suzhou 215123, China.

[8]Réseau sur le Stockage Electrochimique de l'Energie (RS_2E), FR CNRS 3459, France

This Supplementary Information document includes:

Supplementary Figures 1 - 23.

Supplementary Tables 1 and 2.

Supplementary References

1. Characterization of pristine materials

To clarify the difference of surface chemistry between MS-$Ti_3C_2T_x$ and HF-$Ti_3C_2T_x$, TPD-MS, XPS and EDS were performed. According to these characterizations, the content of –Cl is sharply diminished leaving –O the main terminations of MS-$Ti_3C_2T_x$. Thus, the discrepancy in surface chemistry of two MXenes could lie in the different contents of –O terminations.

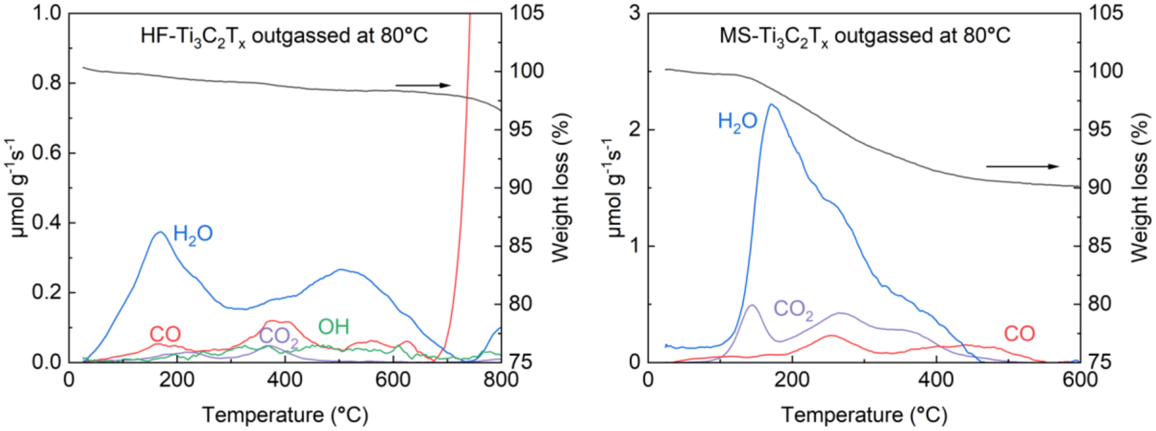

Supplementary Fig. 1. TPD-MS measurements of MS-$Ti_3C_2T_x$ and HF-$Ti_3C_2T_x$. MS-$Ti_3C_2T_x$ shows the absence of –OH terminations and the content of –O terminations is much higher than that of HF-$Ti_3C_2T_x$. The content of –OH in the HF-MXene was calculated as 0.2 wt.%.

Supplementary Table 1. Compositions of MS-$Ti_3C_2T_x$ and HF-$Ti_3C_2T_x$ via EDS analysis. The composition of MS-$Ti_3C_2T_x$ is $Ti_3C_{1.92}O_{2.07}Cl_{0.15}$. MS-$Ti_3C_2T_x$ is mainly terminated with –O terminations with slight content of –Cl terminations and the content of O is higher than that of HF-$Ti_3C_2T_x$.

at.%	Ti	C	O	F	Cl	Al
MS-$Ti_3C_2T_x$	41.95	26.87	28.95	0	2.10	0.13
HF-$Ti_3C_2T_x$	40.41	25.57	19.83	14.19	0	0

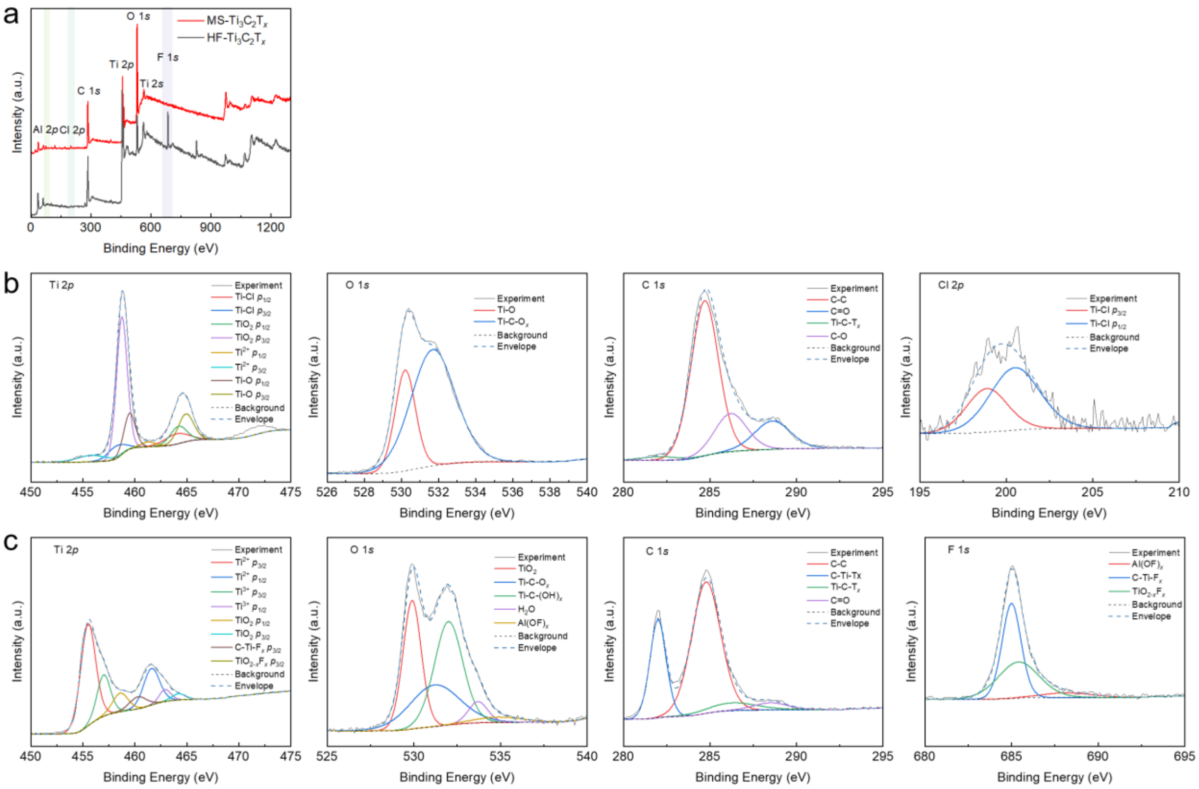

Supplementary Fig. 2. The XPS depth profiles with the depth of 1 μm of pristine MS-$Ti_3C_2T_x$ and HF-$Ti_3C_2T_x$. (a) The global view of XPS spectra. XPS analysis of **(b)** MS-$Ti_3C_2T_x$ and **(c)** HF-$Ti_3C_2T_x$. The ratio of –O to –Cl is around 6.3: 1 (at.%) of MS-$Ti_3C_2T_x$ while the contents of –O, –OH and –F are comparable of HF-$Ti_3C_2T_x$ (around 1: 1.4: 1.4, at.%). The content of –O of MS-$Ti_3C_2T_x$ is much higher than that of HF-$Ti_3C_2T_x$, consistent with the results of EDS and TPD-MS.

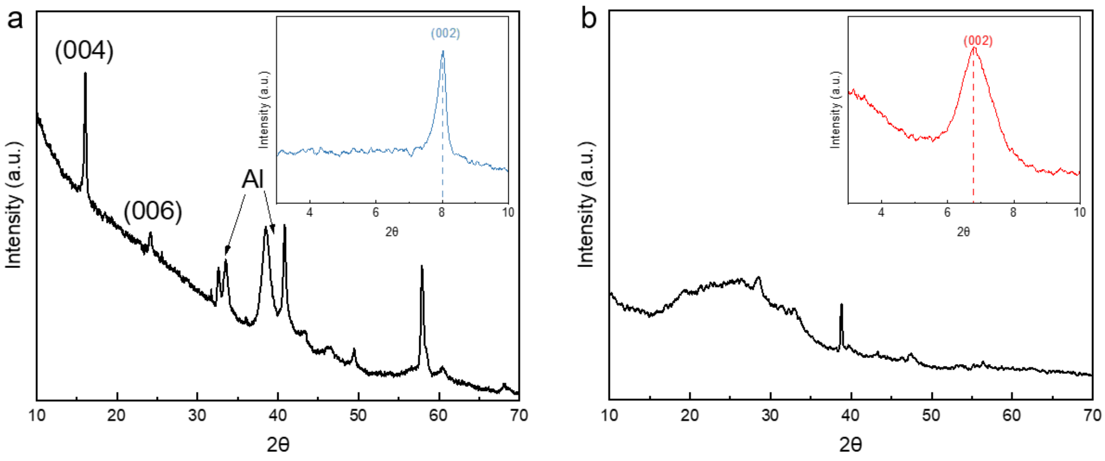

Supplementary Fig. 3. XRD analysis of (a) MS-$Ti_3C_2T_x$ and **(b)** HF-$Ti_3C_2T_x$ illustrate that the *d*-spacing of MS-$Ti_3C_2T_x$ and HF-$Ti_3C_2T_x$ is 1.1 and 1.3 nm, respectively.

2. Electrochemical characterizations of the samples in 1 M LiPF6 (in 1:1 vol/vol ethylene carbonate/dimethyl carbonate, LP30) electrolyte.

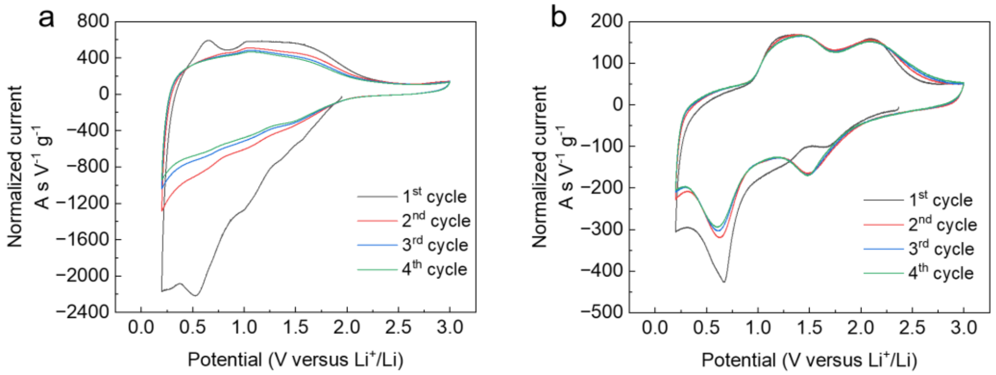

Supplementary Fig. 4. CV tests of the first four cycles at 0.5 mV s^{-1}of (a) MS-$Ti_3C_2T_x$ and (b) HF-$Ti_3C_2T_x$. The formation of the SEI layer in the initial cycle of both MXenes might lead to the low Coulombic efficiency (CE) and the values of CE gradually approach 100% during cycling. The value of CE in the first cycle of MS-$Ti_3C_2T_x$ (51%) is lower than that of HF-$Ti_3C_2T_x$ (73%) which might be attributed to more electrolyte consumption to form the thicker SEI layer.

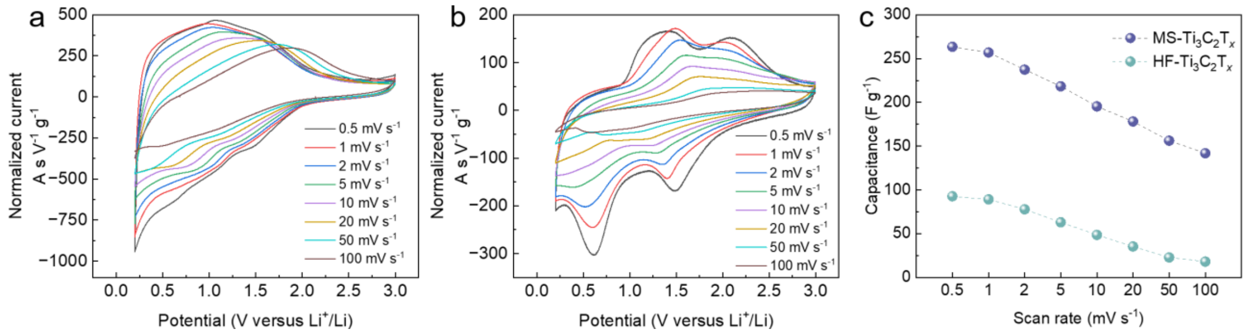

Supplementary Fig. 5. Electrochemical evaluation. CV tests at the scan rates from 0.5 to 100 mV s^{-1} of **(a)** MS-$Ti_3C_2T_x$ and **(b)** HF-$Ti_3C_2T_x$. **(c)** The rate performance of MS-$Ti_3C_2T_x$ and HF-$Ti_3C_2T_x$ in the range of 0.5 to 100 mV s^{-1}.

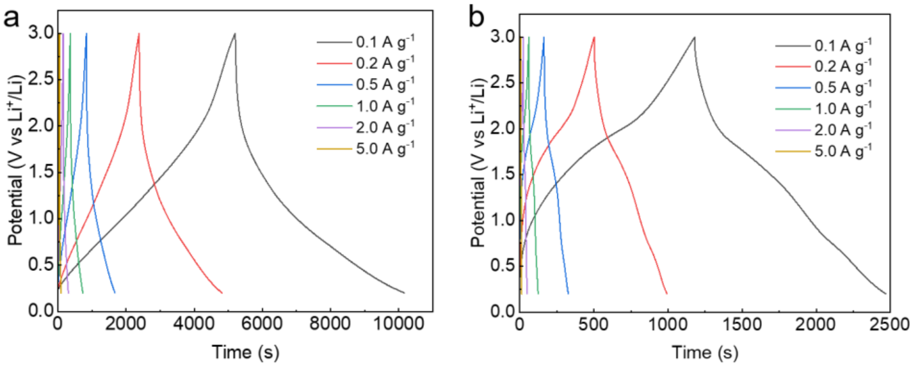

Supplementary Fig. 6. GCD curves at the current densities from 0.1 to 5 A g^{-1} of (a) MS-$Ti_3C_2T_x$ and (b) HF-$Ti_3C_2T_x$. The nonlinear GCD curves without the plateau illustrate both MS- and HF- $Ti_3C_2T_x$ electrodes behave pseudocapacitively.

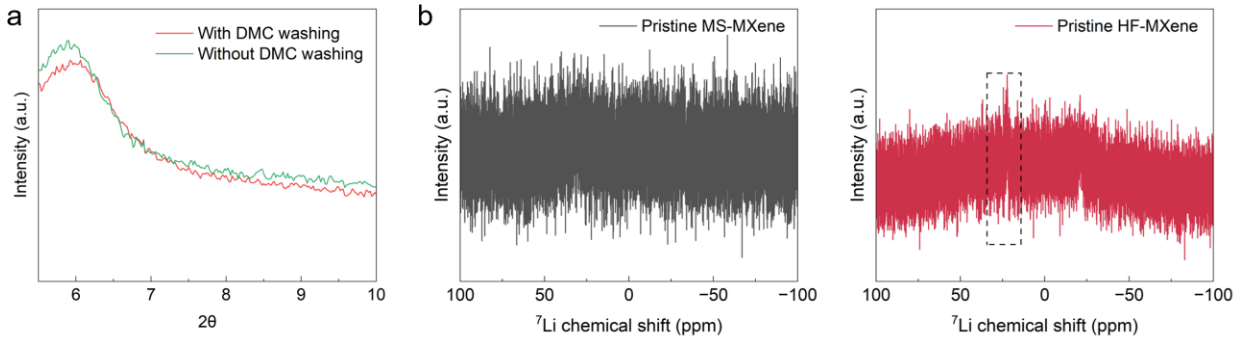

Supplementary Fig. 7. (a) XRD measurements on cycled-HF-MXene samples with and without DMC washing. These two samples were all dried in vacuum overnight. The *d*-spacing almost stayed constant indicating that DMC washing will not affect the solvents in the interlayers. **(b)** ^{7}Li ssNMR spectra of pristine MS-MXene and pristine HF-MXene powders. No Li peaks before Li ions insertion in MS-MXene. As for HF-MXene, the slight peak that appears at 23 ppm (black frame) could be assigned to few Li ions inserted in between interlayers during the LiF + HCl etching process. Notably, the intensity of this weak peak is much lower than the peak for intercalated Li ions for lithiated HF-MXene samples, illustrating the influence of pre-inserted Li ions is negligible.

3. iDPC-STEM and EELS-STEM characterizations of the samples

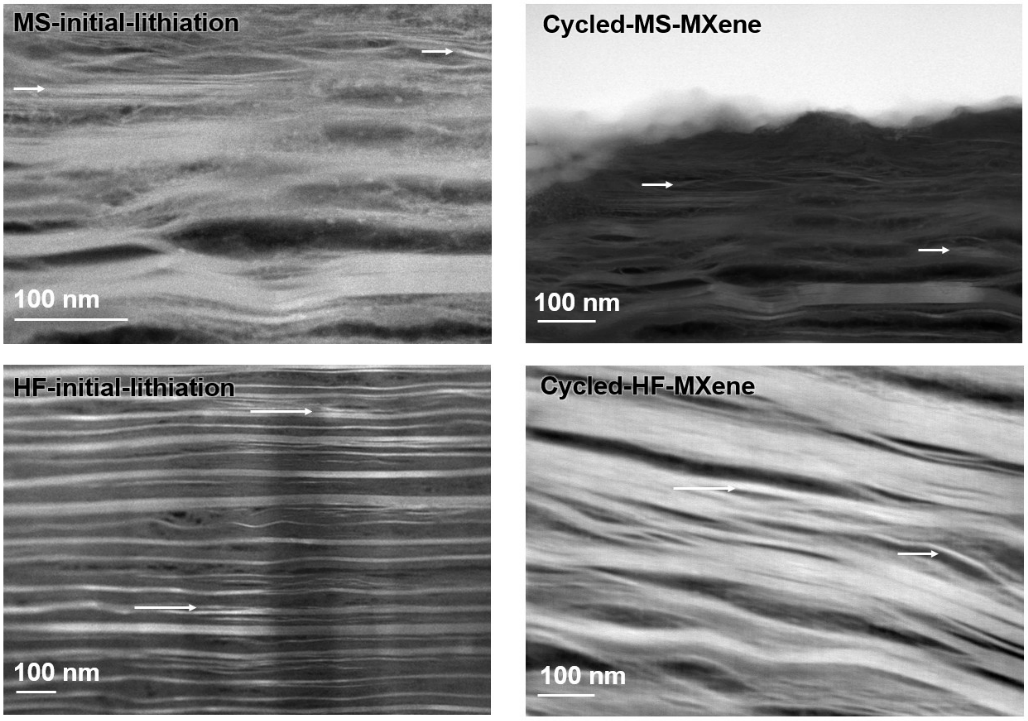

Supplementary Fig. 8. The HAADF-STEM images with low magnification of the FIB milling slices of MS-initial-lithiation, cycled-MS-MXene, HF-initial-lithiation and cycled-HF-MXene samples. The layers are bent in certain areas induced by FIB milling. The bright white ribbons marked by white arrows are the regions with the appropriate zone axis which could be imaged in iDPC-STEM mode.

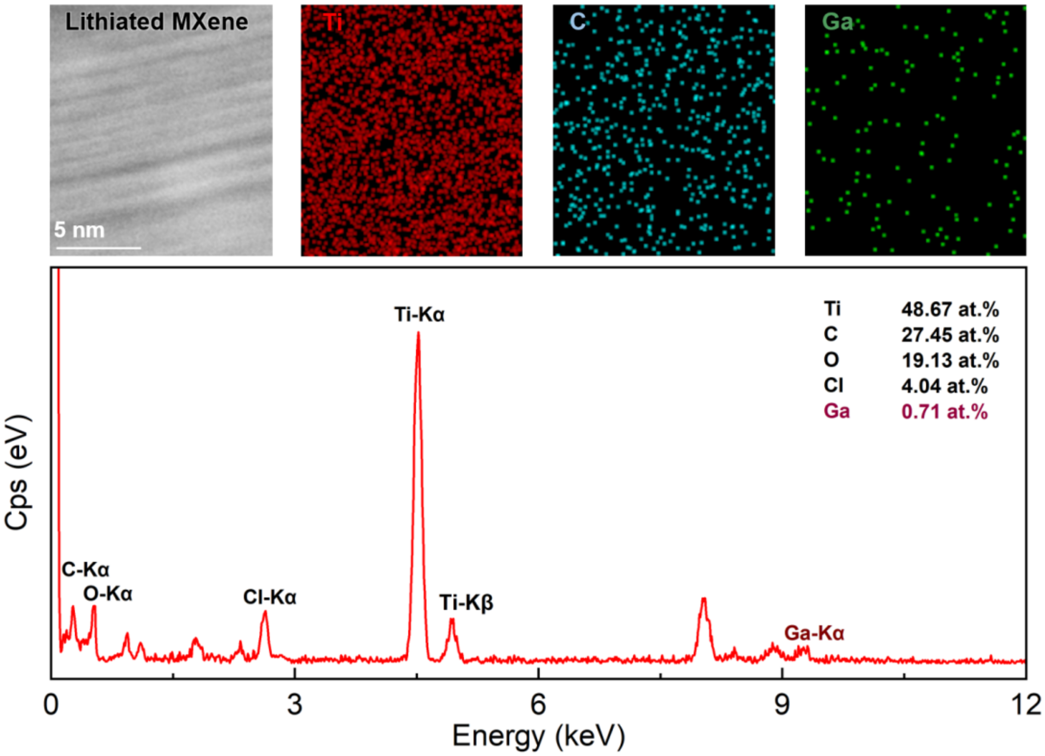

Supplementary Fig. 9. The EDS mapping and spectrum of lithiated MS-MXene FIB milling sample. The much weaker peak of Ga compared to the characteristic peaks of MS-MXene (Ti, C, O and Cl) indicates the negligible effect of Ga ions on the samples.

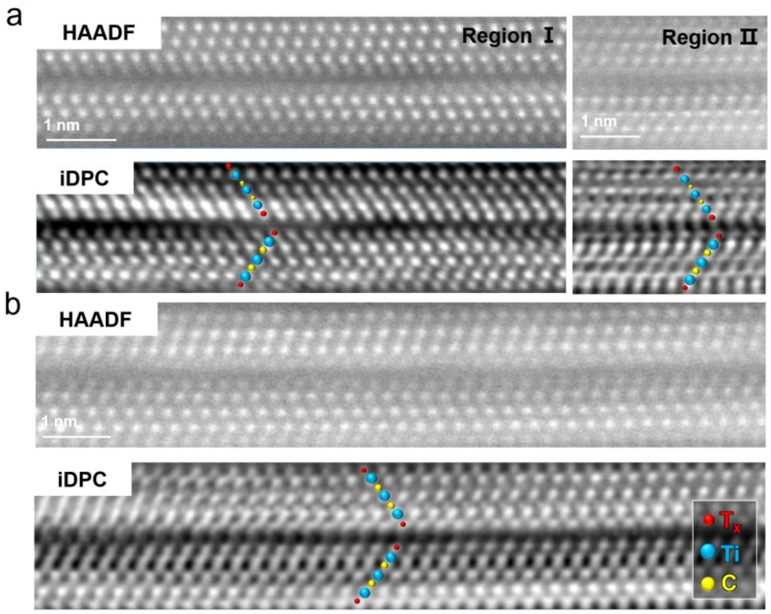

Supplementary Fig. 10. Atomic-scale characterizations of ionic structures after electrochemical polarization of MS-MXenes. The HAADF-STEM images and corresponding iDPC-STEM images at the lower magnification with different regions of **(a)** MS-initial-lithiation and **(b)** cycled-MS-MXene suggesting the consistency of interlayer ionic structures. Ti atoms, C atoms and surface terminations are

marked by blue, yellow and red dots, respectively.

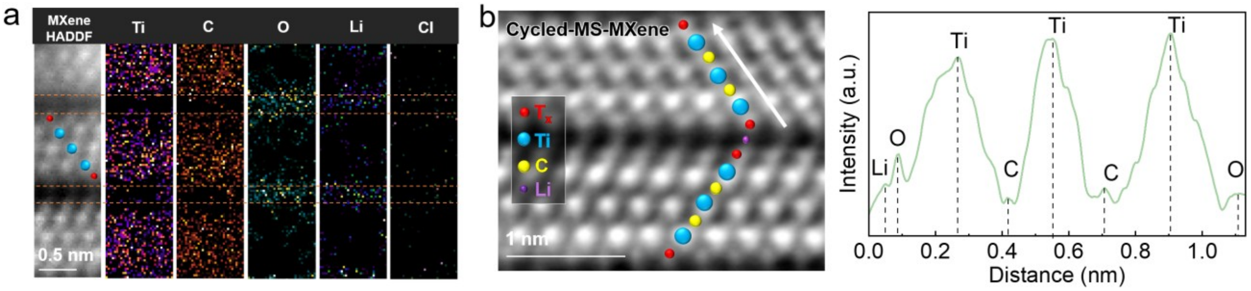

Supplementary Fig. 11. Atomic-scale characterizations of ionic structures after electrochemical polarization. (a) The EELS mapping of Ti, C, O and Li of cycled-MS-MXene sample. Ti atoms and surface terminations are marked by blue and red dots, respectively. Black represents the lowest intensity. HAADF-STEM showcases the 2D projection of the region. As long as there is one Cl atom, the contrast of O terminations will be covered by Cl. **(b)** The intensity profiles along the direction indicated by the white arrow shown in iDPC-STEM image of cycled-MS-MXene sample. Ti atoms, C atoms, surface terminations and Li atoms are marked by blue, yellow, red and purple dots, respectively.

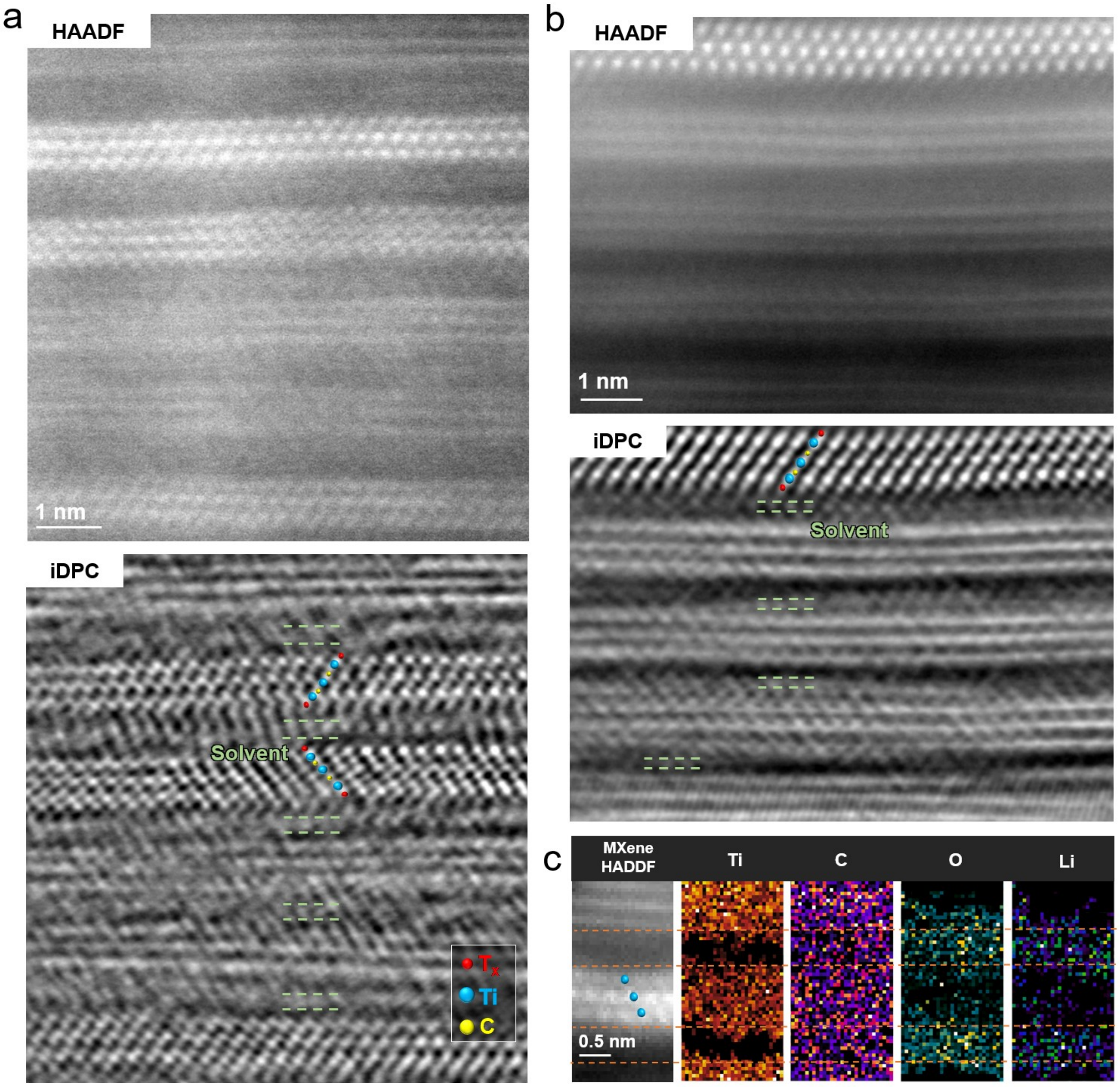

Supplementary Fig. 12. Atomic-scale characterizations of ionic structures after electrochemical polarization of HF-MXenes. The HAADF-STEM images and corresponding iDPC-STEM images at the lower magnification of **(a)** cycled-HF-MXene and **(b)** HF-initial-lithiation suggesting the consistency of interlayer ionic structure. Ti atoms, C atoms and surface terminations are marked by blue, yellow and red dots, respectively. **(c)** The EELS mapping of Ti, C, O and Li of HF-initial-lithiation sample. Ti atoms are marked by blue dots. Black represents the lowest intensity.

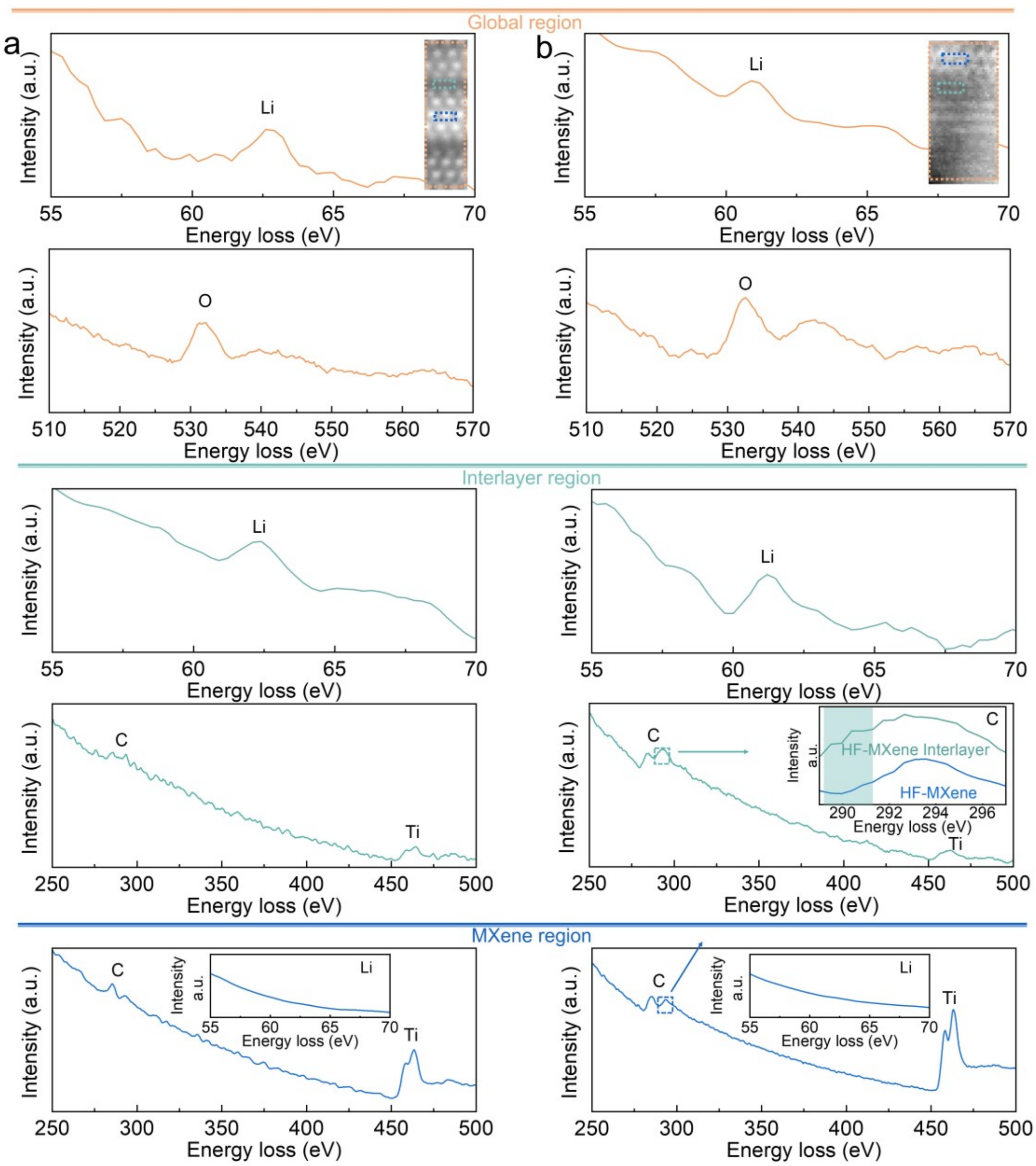

Supplementary Fig. 13. The EELS spectra of lithiated (a) MS-MXene and (b) HF-MXene. The spectra of the global regions were marked as orange lines. The spectra of the interlayer regions were marked as green lines. The spectra of the MXene regions were marked as blue lines.

Obvious peaks of Li illustrated the intercalation of Li ions[1,2]. When we scan MXene (blue frame) region only, characteristic peaks of Ti and C are obvious and similar to the pristine MXenes. Importantly, there is no signal corresponding to Li observed. When the scan region moved to the interlayer (green frame), Li signal could be observed and the Ti peak declines sharply in both MXenes as expected for clean interlayers. The Li signal is weak since the sample is quite thin and the amount of Li ions is not expected to be important (see electrochemical results as well). In

addition, the C peak disappears in MS-MXene while C peak maintains in HF-MXene, indicating the intercalation of carbonate solvents in HF-MXene. When comparing the C peak in the interlayers of HF-MXene with C peak in HF-MXene, the peak at 293 eV in the interlayer is much wider with an extra shoulder in the range of 289 – 292 eV, as shown in the green region in the inset. It may be attributed to the carbonate peak integration at around 290 eV, also verifying the intercalation of carbonate solvents, in line with other studies[3-5].

4. Characterizations of Li ions

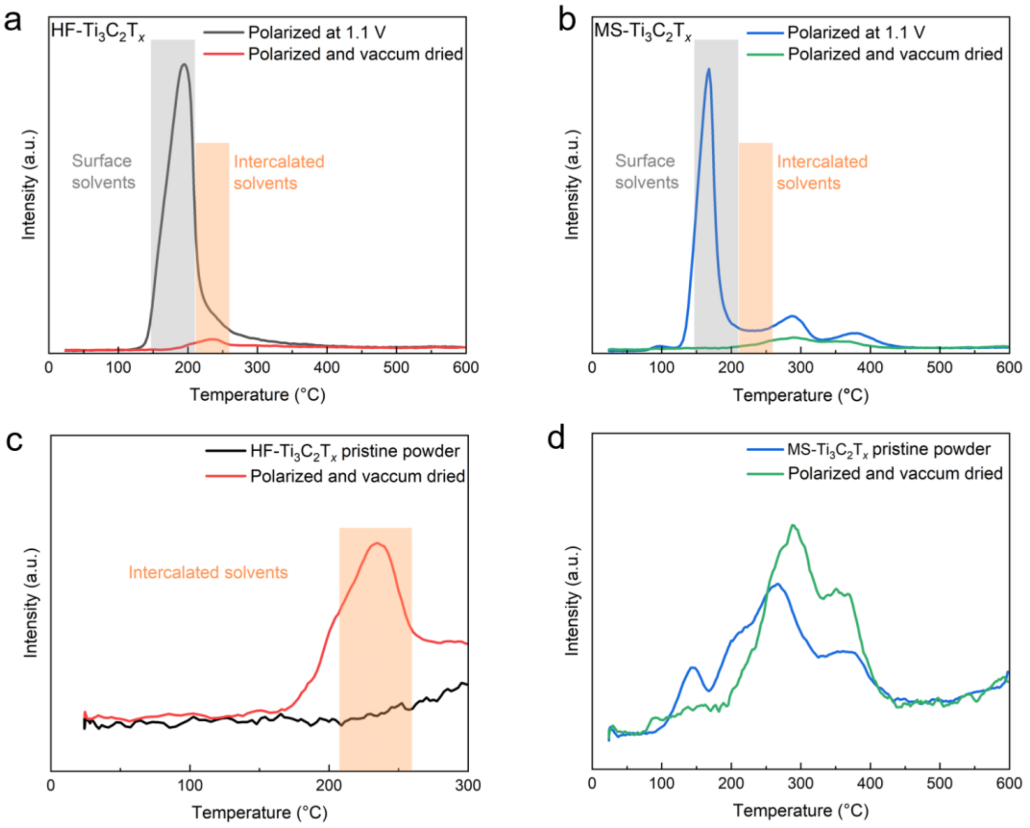

Supplementary Fig. 14. TPD-MS measurement of EC component (m/z = 43) of (a) HF-Ti_3C_2 electrodes polarized at 1.1 V and further vacuum dried for 80°C, **(b)** MS-Ti_3C_2 electrodes polarized at 1.1 V and further vacuum dried for 80°C, **(c)** expanded graph of vacuum dried HF electrodes and pristine HF powder, and **(d)** expanded graph of vacuum dried MS electrodes and pristine MS powder. The intense peak observed between 130 – 200°C for both lithiated MXene samples which disappears after drying could be assigned to the free and surface-adsorbed solvents. After vacuum drying, a significant peak which is visible for HF-Ti_3C_2 at higher temperature (200 – 250°C) but absent for MS-Ti_3C_2 and pristine HF-Ti_3C_2 powders is associated with the solvents intercalated in between MXene interlayers. The absence of the peak for the MS-Ti_3C_2 in the same temperature range indicates that Li ions are inserting the interlayer desolvated.

5. Analysis of the SEI layers formed on the HF- and MS-MXene samples

EDS, HRTEM[6], XPS[7], TOF-SIMS[8] and FTIR[9] techniques were used to further analyze the SEI layers (see Supplementary Figs. 15-21). According to the HRTEM images (Supplementary Figs. 16 and 17), a variety of lattice fringes with distinct distances corresponding to different

inorganic components (e.g. Li_2CO_3, Li_2O, LiF) suggests that the SEI layer formed on MS-$Ti_3C_2T_x$ is dense and stable. Moreover, the presence of organic components is mainly found on the outer layer of SEI in contact with the electrolyte via XPS, TOF-SIMS and FTIR (see Supplementary Figs. 18-21). After cycling, the contents of Li_2CO_3 and F-contained inorganic components increase for MS-MXene while the organic components and Li_2O decrease. Differently, LiF is the main inorganic component of SEI in HF-$Ti_3C_2T_x$ confirming the results of a recent sudy[10]. The presence of more organic components in SEI of HF-$Ti_3C_2T_x$ than MS-$Ti_3C_2T_x$ may explain the degradation of the SEI layer during GCD cycling by a slow but continuous reduction of the solvents on the MXene surface, which further supports some recent results of the literature[10]. It can be hypothesized that more content of –O terminations of MS-$Ti_3C_2T_x$ results in the formation of more O-contained inorganic components in SEI whose energy barriers of Li ions diffusion are lower than that of LiF[11].

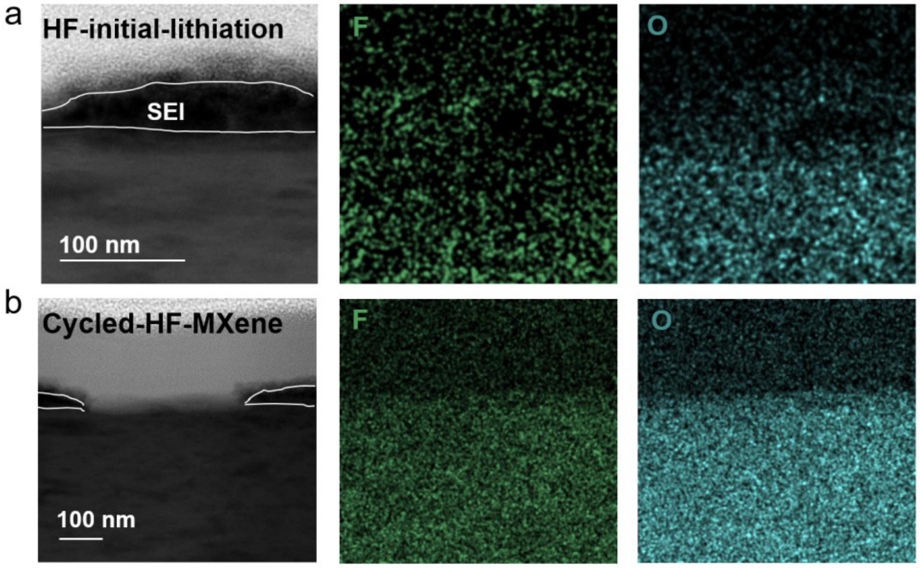

Supplementary Fig. 15. EDS mapping of the cross section of (a) HF-initial-lithiation and (b) cycled-HF-MXene illustrating the degradation of SEI layers.

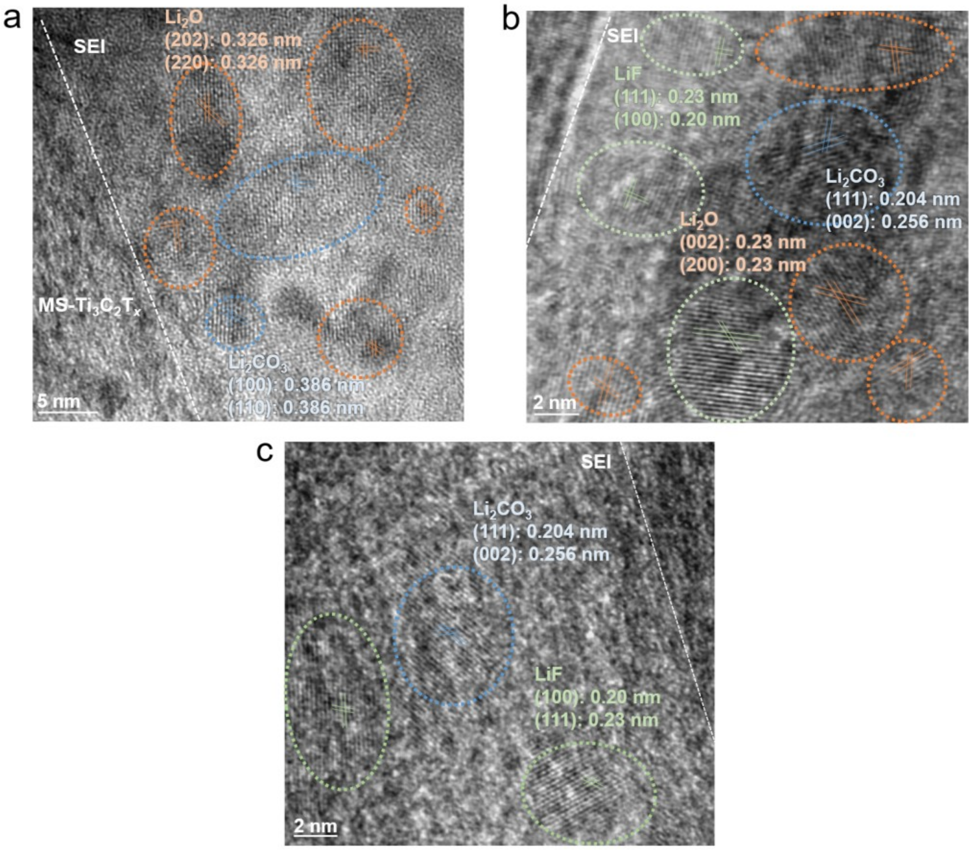

Supplementary Fig. 16. The HRTEM images of the MXene/SEI layer interface of **(a)** Initial cycle via CV test of MS-$Ti_3C_2T_x$. **(b)** The third cycle via CV test of MS-$Ti_3C_2T_x$. **(c)** Initial cycle via CV test of HF-$Ti_3C_2T_x$.

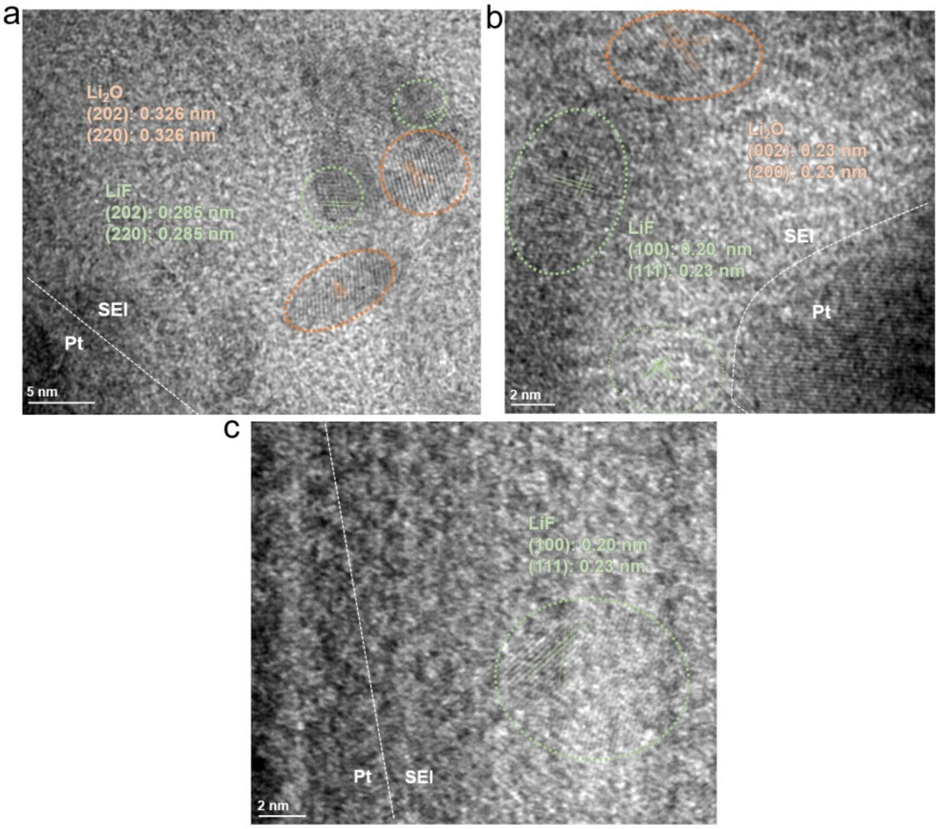

Supplementary Fig. 17. The HRTEM images of the SEI layer/electrolyte interface of **(a)** Initial cycle via CV test of MS-$Ti_3C_2T_x$. **(b)** The third cycle via CV test of MS-$Ti_3C_2T_x$. **(c)** Initial cycle via CV test of HF-$Ti_3C_2T_x$ of outer layer in SEI layer.

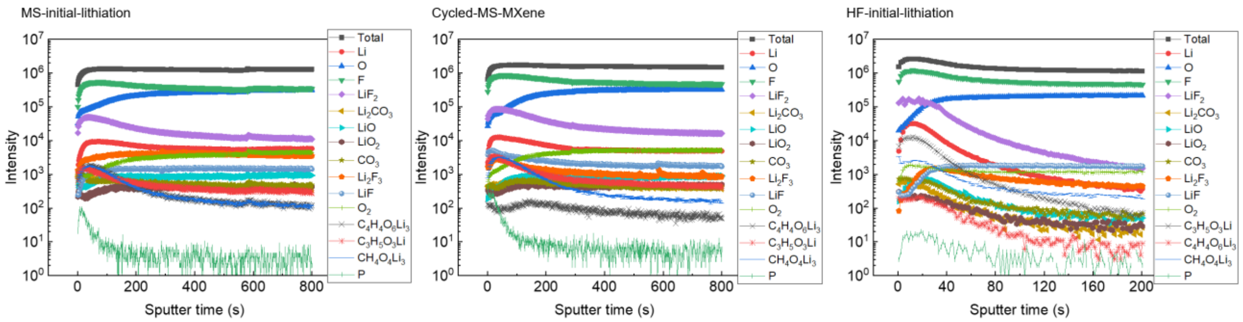

Supplementary Fig. 18. Depth profiles in negative mode of the SEI layers in three conditions via TOF-SIMS. The contents of organic components and inorganic components exhibit different evolutions when the depth increases. The negligible signals of P indicate that washing by DMC could effectively remove the electrolytes in the surface of the lithiated MXene electrodes.

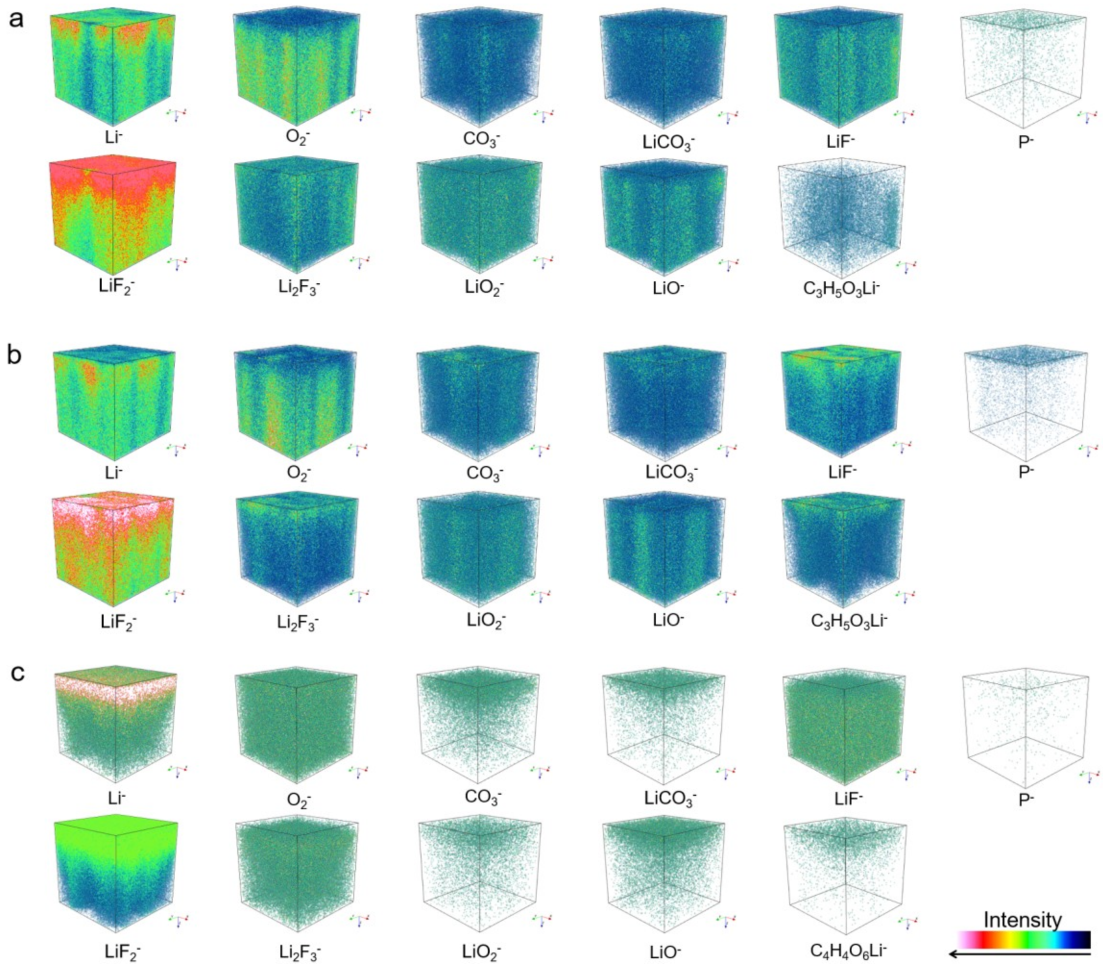

Supplementary Fig. 19. 3D reconstructions of different components in SEI layers in three conditions via TOF-SIMS. **(a)** Initial cycle via CV test of MS-$Ti_3C_2T_x$. **(b)** The third cycle via CV test of MS-$Ti_3C_2T_x$. **(c)** Initial cycle via CV test of HF-$Ti_3C_2T_x$. Organic components mainly distribute in the outer layer of SEI in MS-$Ti_3C_2T_x$ and HF-$Ti_3C_2T_x$. The contents of F-contained inorganic components and Li_2CO_3 of SEI in MS-$Ti_3C_2T_x$ increase while the content of organic components decreases after cycling. Less O-contained inorganic components are detected in HF-$Ti_3C_2T_x$ than MS-$Ti_3C_2T_x$. The negligible signals of P indicate that washing by DMC could effectively remove the electrolytes in the surface of the lithiated MXene electrodes.

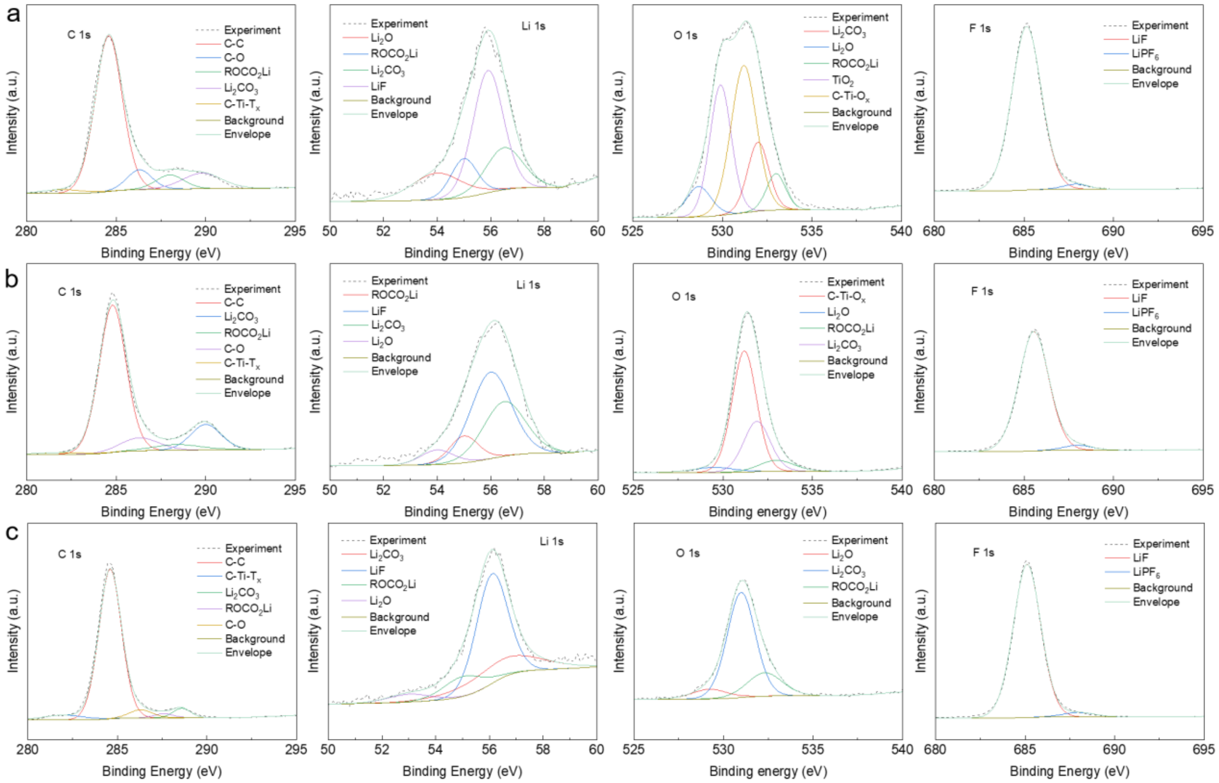

Supplementary Fig. 20. XPS analysis of SEI layers in three conditions. **(a)** Initial cycle via CV test of MS-$Ti_3C_2T_x$. **(b)** The third cycle via CV test of MS-$Ti_3C_2T_x$. **(c)** Initial cycle via CV test of HF-$Ti_3C_2T_x$. The results show less O-contained components and more organic components in SEI of HF-$Ti_3C_2T_x$ than MS-$Ti_3C_2T_x$. After cycling, the contents of Li_2O and organic components decrease and the content of Li_2CO_3 improves in SEI of MS-$Ti_3C_2T_x$.

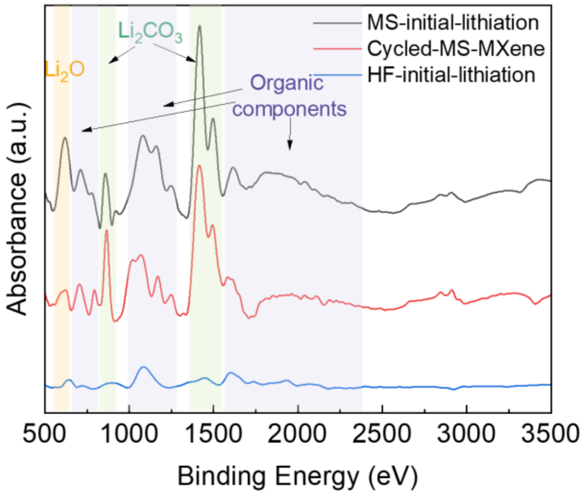

Supplementary Fig. 21. FTIR analysis of SEI layers in three conditions. More Li_2O and Li_2CO_3 in SEI of MS-$Ti_3C_2T_x$ than HF-$Ti_3C_2T_x$. After cycling, the contents of Li_2O and organic components decrease and the content of Li_2CO_3 improves in SEI of MS-$Ti_3C_2T_x$.

6. MD simulations for the ionic structures in the interlayers of HF- and MS-MXene samples

As illustrated in Supplementary Figs. 22 and 23 and Supplementary Videos 1 and 2, simulations reveal that only desolvated Li ions can intercalate into the interlayers of negatively charged MS-MXene, whereas solvent molecules intercalate together with Li ions into HF-MXene. The coordination number of CH_3 at the MXene surface, close to different surface terminations, was calculated based on the results of inset in Supplementary Fig. 23a. The RDF cutoff radius for defining the CH_3-O and CH_3-F coordination numbers in the absence of confinement were taken as the first minimum of the radial distribution function shown in the inset of Supplementary Fig. 23a, that is around 3.5 Å. This value of 3.5 Å was further used to calculate the coordination number of CH_3 around -O and -F terminations under confinement (Supplementary Fig. 23a). About 2.00 CH_3 groups are coordinated near –F groups, while around 1.58 CH_3 molecules are associated with oxygen-containing terminations, indicating a stronger F–CH_3 interaction. MD results also reveal that the distance between intercalated Li ions and -O terminations is about 0.3 Å closer in MS-MXene compared to HF-MXene (Supplementary Fig. 23c). Together with the formation of a stable SEI layer onto MS-MXene, the high affinity of the CH_3 groups from the electrolyte solvent for the F-termination groups of the HF-MXene well supports the difference in electrochemical behaviors between the two MXenes. The detailed force field for MS-MXene and HF-MXene models in our simulations is summarized in Supplementary Table 2.

Supplementary Table 2. Lennard-Jones coefficients for the MS-MXene and HF-MXene layer with -F, =O, -OH, -Cl terminations.

Element	ε / kcal mol^{-1}	σ / nm
Ti	0.60870	0.19565
C	0.06600	0.35000
F	0.16730	0.31430
O(=O)	0.15540	0.31656
O(-OH)	0.18480	3.55320
H(-OH)	0.01000	0.90000
Cl	0.12470	3.78500

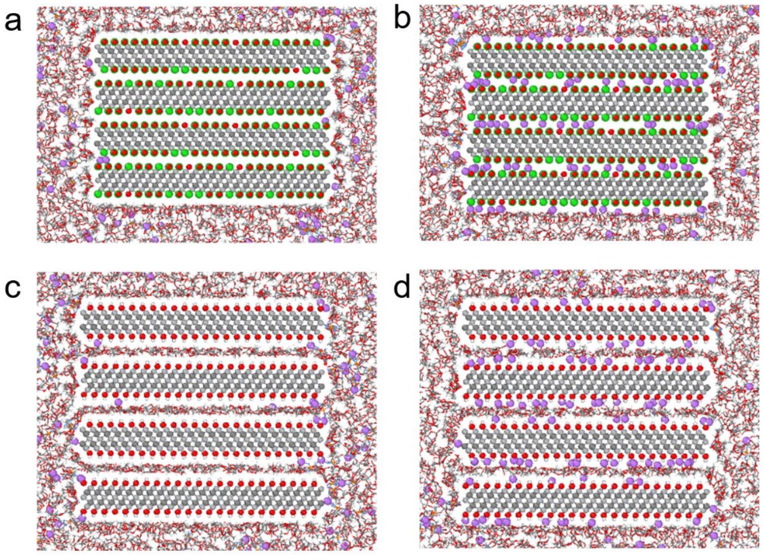

Supplementary Fig. 22. Snapshot of the MD simulation model for MS-MXene electrode in LP30 electrolyte at **(a)** equilibrium state and **(b)** charged state, and HF-MXene electrode at **(c)** equilibrium state and **(d)** charged state.

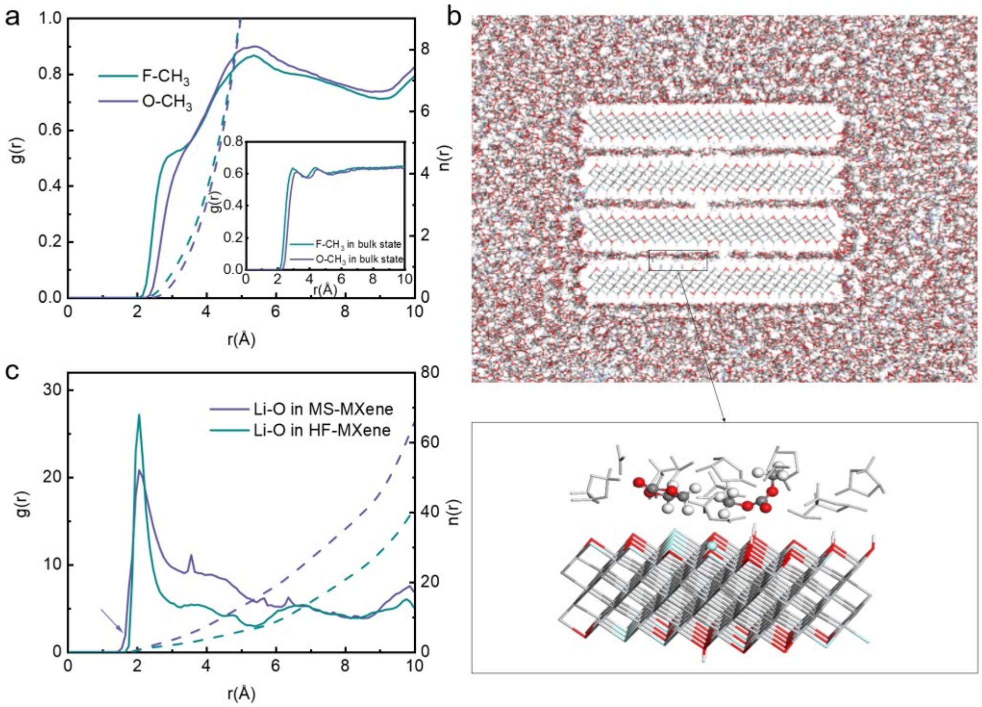

Supplementary Fig. 23. (a) The calculated radial distribution function (RDF) of MXene surface terminations F and O with CH_3 of intercalated solvent in HF-MXene. Inset gives the calculated radial distribution function of F-CH_3 and O-CH_3 in case of single-layer MXene in LP30 electrolyte with absence of confinement. **(b)** Snapshot of the solvent intercalated in HF-MXene layers. **(c)** The calculated radial distribution function of intercalated Li ions with O terminations of MS-MXene and HF-MXene, respectively.

Supplementary References

1 Zhang, W. *et al.* Kinetic pathways of ionic transport in fast-charging lithium titanate. *Science* **367**, 1030-1034 (2020).

2 Saitoh, M. *et al.* Systematic analysis of electron energy-loss near-edge structures in Li-ion battery materials. *Phys. Chem. Chem. Phys.* **20**, 25052-25061 (2018).

3 Zhang, Z. *et al.* Cathode-electrolyte interphase in lithium batteries revealed by cryogenic electron microscopy. *Matter* **4**, 302-312 (2021).

4 Huang, W. *et al.* Dynamic structure and chemistry of the silicon solid-electrolyte interphase visualized by cryogenic electron microscopy. *Matter* **1**, 1232-1245 (2019).

5 Yin, Z.-W. *et al.* Advanced electron energy loss spectroscopy for battery studies. *Adv. Funct. Mater.* **32**, 2107190 (2022).

6 Wi, T.-U. *et al.* Revealing the dual-layered solid electrolyte interphase on lithium metal anodes via cryogenic electron microscopy. *ACS Energy Lett.* **8**, 2193-2200 (2023).

7 Chen, J. *et al.* Electrolyte design for LiF-rich solid–electrolyte interfaces to enable high-performance microsized alloy anodes for batteries. *Nat. Energy* **5**, 386-397 (2020).

8 Ma, C., Xu, F. & Song, T. Dual-layered interfacial evolution of lithium metal anode: SEI analysis via TOF-SIMS technology. *ACS Appl. Mater. Interfaces* **14**, 20197-20207 (2022).

9 Ye, J.-Y., Jiang, Y.-X., Sheng, T. & Sun, S.-G. In-situ FTIR spectroscopic studies of electrocatalytic reactions and processes. *Nano Energy* **29**, 414-427 (2016).

10 Sunny, S., Coppel, Y., Taberna, P. L. & Simon, P. Characterization by NMR spectroscopy of the SEI layer formed on Ti_3C_2 MXene materials prepared with various terminations. *J. Electrochem. Soc.* **171**, 030512 (2024).

11 Tan, J., Matz, J., Dong, P., Shen, J. & Ye, M. A growing appreciation for the role of LiF in the solid electrolyte interphase. *Adv. Energy Mater.* **11**, 2100046 (2021).
