# Supplementary material for: Ion desolvation for boosting the charge storage performance in Ti3C2 MXene electrode
Source: Nat Commun. 2025 Apr 23;16:3813. doi: 10.1038/s41467-025-58700-x (PMC12018951; doi:10.1038/s41467-025-58700-x)
Supplement: Supplementary file 2 — Description of Additional Supplementary Files [file 41467_2025_58700_MOESM2_ESM.pdf]

## Description of Additional Supplementary Files

Supplementary Video 1. The MD simulation model for Li ions inserted into the HF-MXene electrode.

Supplementary Video 2. The MD simulation model for Li ions inserted into the MS-MXene electrode.
